# BET-Inhibitor I-BET762 and PARP-Inhibitor Talazoparib Synergy in Small Cell Lung Cancer Cells

**DOI:** 10.3390/ijms21249595

**Published:** 2020-12-16

**Authors:** Francesco Paolo Fiorentino, Irene Marchesi, Christoph Schröder, Ronny Schmidt, Jun Yokota, Luigi Bagella

**Affiliations:** 1 Department of Biomedical Science, and National Institute of Biostructures and Biosystems, University of Sassari, 07100 Sassari, Italy; fpfiorentino@kitosbiotech.org; 2 Kitos Biotech Srls, Porto Conte Ricerche, 07041 Alghero, Italy; imarchesi@kitosbiotech.org; 3 Sciomics GmbH, 69151 Neckargemünd, Germany; schroeder@sciomics.de (C.S.); ronny.schmidt@sciomics.de (R.S.); 4 Division of Genome Biology, National Cancer Center Research Institute, Chuo-ku, Tokyo 104-0045, Japan; jyokota-catv@j05.itscom.net; 5 Center for Biotechnology, Department of Biology, Sbarro Institute for Cancer Research and Molecular Medicine, College of Science and Technology, Temple University, Philadelphia, PA 19122, USA

**Keywords:** SCLC, BET, PARP, BMN673, GSK-525762A

## Abstract

Small cell lung cancer (SCLC) is an aggressive type of lung cancer with high mortality that is caused by frequent relapses and acquired resistance. Despite that several target-based approaches with potential therapeutic impact on SCLC have been identified, numerous targeted drugs have not been successful in providing improvements in cancer patients when used as single agents. A combination of targeted therapies could be a strategy to induce maximum lethal effects on cancer cells. As a starting point in the development of new drug combination strategies for the treatment of SCLC, we performed a mid-throughput screening assay by treating a panel of SCLC cell lines with BETi or AKi in combination with PARPi or EZH2i. We observed drug synergy between I-BET762 and Talazoparib, BETi and PARPi, respectively, in SCLC cells. Combinatorial efficacy was observed in *MYCs*-amplified and *MYCs*-wt SCLC cells over SCLC cells with impaired MYC signaling pathway or non-tumor cells. We indicate that drug synergy between I-BET762 and Talazoparib is associated with the attenuation HR-DSBR process and the downregulation of various players of DNA damage response by BET inhibition, such as CHEK2, PTEN, NBN, and FANCC. Our results provide a rationale for the development of new combinatorial strategies for the treatment of SCLC.

## 1. Introduction

Small cell lung cancer (SCLC) is the most aggressive type of lung cancer [1,2]. Genome-wide analyses in SCLC specimens confirmed, in ~15% of cases, gene amplification of one of the members of the MYC gene family, *MYC*, *MYCL*, or *MYCN* (hereafter *MYCs*) [3,4]. We previously reported that cell cycle arrest and apoptosis were consequences of MYC inhibition in SCLC cells, which indicated that MYCs could be targets for therapy in a subset of SCLC patients [5].

A pharmacological approach for inhibiting MYCs activity is represented by Bromodomain and Extra-Terminal domain (BET) inhibitors [6]. BET proteins maintain and facilitate oncogenic transcription by recruiting transcriptional machinery or binding to super-enhancers [7,8,9]. The suppression of *MYCs* activities by BET inhibitors (BETi) was reported in various types of tumors, including SCLC [10,11,12]. Furthermore, SCLC cells have been shown to be sensitive to BET inhibition [12,13,14,15,16]. However, several mechanisms of intrinsic drug resistance to BET inhibition were proposed [17]. Another approach for targeting MYC signaling pathway in SCLC cells is represented by Aurora Kinase inhibitors (AKi), which showed elevated toxicity in SCLC cells carrying MYC amplification [3,18,19,20]. Mechanistically, it has been proposed that MYCs activate the transcription of Aurora Kinases, which, in turn, may provide a growth advantage in the absence of p53 [21]. However, a clinical trial that was carried out to evaluate the anti-tumor efficacy of an AKi in solid tumors showed marginal activity in several types of tumors, including SCLC [22]. As reviewed by Lopez and Banerji, a large number of targeted drugs have not been successful in providing improvements in patients with cancer when used as single agents [23]. This is partly due to the intrinsic resistance of tumor cells, which, in some instances, has been successfully overcome by a combined therapy approach [23,24]. 

Poly-(ADP)-ribose polymerase-1 (PARP-1) and Enhancer of Zeste Homolog 2 (EZH2) have been identified as potential therapeutic targets in SCLC [25]. PARP-1 is a DNA damage sensor that binds damaged DNA at single-strand breaks to lead the recruitment of DNA repair effectors, followed by its release from repaired DNA [26,27]. In tumor cells, PARP-1 can prevent cells from apoptosis by cleaving DNA-damage and, thus, inhibiting cell death [27]. PARP inhibitors (PARPi) “trap” PARP-1 on DNA, preventing its release from damaged sites and interfering with its activity [26]. PARP-1 has been reported as highly expressed in SCLC cells and its suppression or pharmacological inhibition has been shown to impair SCLC cell proliferation [25,28,29,30,31,32]. EZH2, which is the catalytic subunit of the “polycomb repressive complex 2” (PRC2), is upregulated in SCLC cells upon the inactivation of the RB-E2F pathway, leading to a silencing of tumour suppressor genes and ultimately promoting SCLC cell proliferation [25,30,33]. The suppression of EZH2 has been shown to promote cell cycle arrest and apoptosis of SCLC cells [25,28,29,30,34]. 

A synergistic effect of EZH2 inhibitors (EZH2i) or PARPi with BETi or AKi on SCLC cell viability is still unknown. Therefore, we aimed to identify an effective drug combination for the treatment of SCLC by evaluating the efficacy of BETi or AKi in combination with EZH2i or PARPi on a panel of SCLC cell lines.

## 2. Results

### 2.1. I-BET762 and Talazoparib Showed a Combinatorial Efficacy on MYCs-Amplified SCLC Cells

A battery of 10 SCLC cell lines were grown as 3D tumor spheroids and treated with eight small molecule inhibitors, two for each target (Table 1 and Table 2), in order to assess the efficacy of BET or Aurora Kinase inhibition in combination with EZH2 or PARP-1 inhibition on SCLC cells [35,36,37,38,39]. Gene amplification and the expression levels of *MYCs* were previously assessed in these SCLC cell lines [4,5]. Two MAX-inactivated SCLC cell lines, Lu134 and Lu165, were included to evaluate treatment sensitivity in a background of *MYCs* impaired ability, since MYCs require heterodimerization with MAX to activate the transcription of their target genes [40,41]. HECV endothelial cell line was added in order to assess drug efficacy on a representative non-tumor spheroid model. 

**Figure 1:**
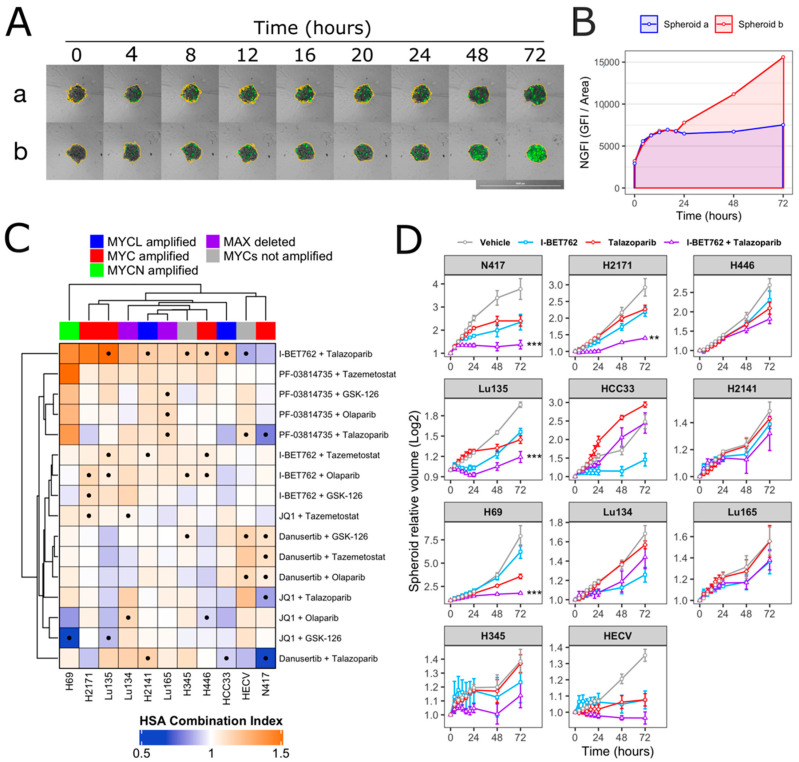
Synergistic efficacy of I-BET762 and Talazoparib on the viability and growth of small cell lung cancer (SCLC) cells. (**A**) Merged brightfield and green fluorescence images of representative Lu135 untreated (a) or I-BET762 and Talazoparib combination-treated (b) spheroids over 72 h of culture. Spheroid edges, shown in yellow, were used to measure spheroid volumes and Green Fluorescence Integral (GFI), representative of relative number of cells and relative number of dead cells, respectively. Bar of 1,000 µM is shown bottom right. (**B**) Normalized GFI of spheroids shown in (A). Area under a curve of normalized GFI (NGFI) was used as a parameter representative of kinetics of cytotoxicity over a period of 72 h. (**C**) Heatmap showing Highest Single Agent Combination Index (HSA-CI) of NGFI, representative of cell death. A black dot inside the squares indicates a Benjamini-Hochberg corrected *p*-value < 0.05. (**D**) Timeplot of volumes of I-BET762 and Talazoparib-treated spheroids (relative to start of treatment). Values shown mean ± SE. ** *p* < 0.01, or *** *p* < 0.001 BH-adjusted HSA Combination Index CDF.

**Figure 2:**
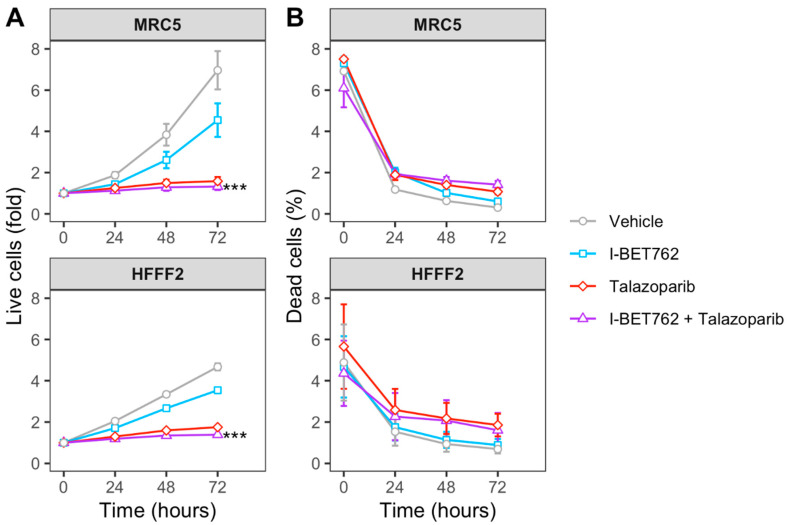
Efficacy of I-BET762 and Talazoparib combination on non-tumor cells. (**A**) Timeplot of live cell number in non-tumor fibroblast cell lines HFFF2 and MRC5 that were treated with I-BET762 and Talazoparib as single agents or as a combination. (**B**) Percentage of dead cells. Values shown mean ± SE. Benjamini-Hochberg corrected one-way ANOVA was performed between Talazoparib-treated and drug combination-treated samples, since Talazoparib as single agent showed increased anti-proliferative activity compared to I-BET762. *** p < 0.001.

Spheroids were treated with each compound as single agent (0.5 µM) or in combination (0.5 µM of each compound). Spheroid volumes, representative of cell number, and green fluorescence intensity (GFI), representative of cell death, were monitored by live-cell imaging immediately after starting the treatment and after 4, 8, 12, 16, 20, 24, 48, and 72 h (Figure 1A). The area under a curve of GFI over time was calculated in order to obtain a value representative of kinetics of cell death (Figure 1B), and the Highest Single Agent (HSA) Combination Index was calculated in order to identify compound combinations with improved cytotoxicity as compared to single agents, termed additive efficacy (Figure 1C) [42]. Among all tested conditions, BET inhibitor GSK525762 (I-BET762) showed a significant additive effect with PARP inhibitor BMN-673 (Talazoparib) in four out of seven tested *MYCs*-amplified SCLC cell lines, Lu135, H2141, H446, and HCC33 (Figure 1C and Figure S1). An additive cytotoxic efficacy by this drug combination was also observed in H345 SCLC cells, which do not carry amplification of any of the MYCs, but showed high levels of MYCL (Figure 1C) [12]. In the remaining *MYCs*-amplified SCLC cell lines, N417, H2171, and H69, I-BET762 and Talazoparib showed additive efficacy in reducing spheroid growth (Figure 1D). In a similar manner, a combination of I-BET762 and Olaparib, the remaining tested PARP inhibitor, showed higher cytotoxicity in H2171, H446, Lu135, and H345 SCLC cells when compared to single agents, confirming the additive effect by I-BET762 with PARP inhibition (Figures S2 and S3). Because the absence of significant additive effects was observed by combining I-BET762 and PARP inhibitors in the two MAX-deleted SCLC cell lines, Lu134 and Lu165, our results indicate that these investigational drugs have a preferential or selective combinatorial efficacy on SCLC cells with an active MYC signaling pathway. Interestingly, Talazoparib showed reduced toxicity and an improved, non-significant, cytostatic effect in non-tumor HECV spheroids when in combination with I-BET762 (Figure 1C,D). No additive effects were observed in cells that were treated with JQ1, the remaining tested BET inhibitor, in combination with PARP inhibitors. However, it is likely that drug synergy could not be assessed in JQ1-treated spheroids due to the high cytotoxicity displayed by this molecule as a single agent in SCLC spheroids (Figures S4 and S5). To confirm the efficacy of I-BET762 and Talazoparib combination on non-tumor cells other than HECV, lung MRC5 and foreskin HFFF2 fibroblasts were grown as monolayers, since these cell lines do not proliferate as three-dimensional (3D) spheroids, and they were treated with the two investigational drugs as single agents or in combination at the same concentrations that were used in the screenings. The cells were monitored by live-imaging in order to assess the number of live and dead cells at 24, 48, and 72 h of treatment. A strong cytostatic effect was observed by Talazoparib in both non-tumor cell lines, which was slightly increased by combination with I-BET762, as shown in Figure 2A. No cytotoxic effects were observed at all of the tested conditions (Figure 2B).

Overall, these results indicate that BET inhibitor I-BET762 and PARP inhibitors have a preferential or selective combinatorial efficacy on *MYCs*-amplified or *MYCs*-wt SCLC cells over non-tumor cells or SCLC cells with an impaired MYC signaling pathway. Furthermore, I-BET762 showed additive cytostatic or cytotoxic efficacy in combination with PARP inhibitor Talazoparib in all of the tested SCLC cell lines with an intact MYC signaling pathway.

### 2.2. Combinatorial Treatment of I-BET762 and Talazoparib at Clinically Relevant Concentrations Showed Increased Efficacy on MYCs-Amplified SCLC Cell Growth

**Figure 3:**
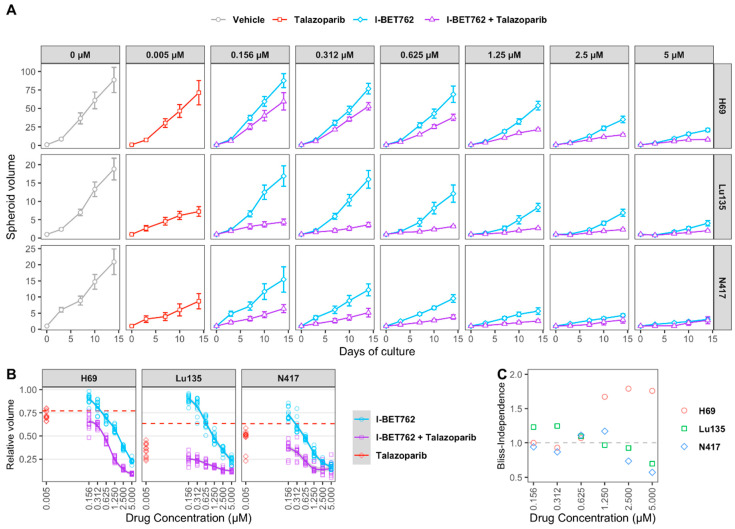
Efficacy of I-BET762 and Talazoparib on long-term growth of SCLC cells. (**A**) Timeplot of H69 (**A**), Lu135 (**B**), or N417 (**C**) spheroid volumes treated with Talazoparib (5 nM) or I-BET762 (0.156 µM, 0.312 µM, 0.625 µM, 1.25 µM, 2.5 µM or 5 µM) as single agents or as a combination. Values shown are mean ± sd of 12 replicates. (**B**) Volumes of treated spheroids after 14 days of treatment normalized to volumes in untreated spheroids at indicated concentrations. Volumes of Talazoparib-treated spheroids is shown as red dashed lines. (**C**) Bliss Independence Combination Index at each concentration of I-BET762 combined with Talazoparib 5 nM after 14 days of treatment.

H69, Lu135, and N417 spheroids were treated with six concentrations of I-BET762 ranging from 5 µM to 160 nM as single agent or in combination with 5 nM Talazoparib in order to confirm drug synergy between I-BET762 and Talazoparib. Spheroids were maintained in culture for 14 days by replacing medium twice per week and their volume was monitored by live-imaging. The combined treatment of the two investigational drugs reduced spheroid growth in all of the examined cell lines at all tested concentrations (Figure 3A and Table 3). Bliss Independence Combination Index was calculated in order to assess a synergistic effect [42]. Because the Bliss Independence model only applies to effects that are expressed as probabilities ranging between 0 and 1, volumes of treated spheroids were first normalized to the mean volume of untreated ones (Figure 3B). A synergistic efficacy was observed at higher concentrations of I-BET762 in H69 (5 µM, 2.5 µM, 1.25 µM), lower concentrations in Lu135 (625 nM, 312 nM, 156 nM), and at 0.625 nM and 0.312 nM in N417, as shown in Figure 3C. Our results confirmed a synergistic efficacy between I-BET762 and Talazoparib in *MYCs*-amplified SCLC cells. 

### 2.3. I-BET762 Treatment Induces Apoptosis and Down-Regulation of Double-Strand Break Repair Proteins

**Figure 4:**
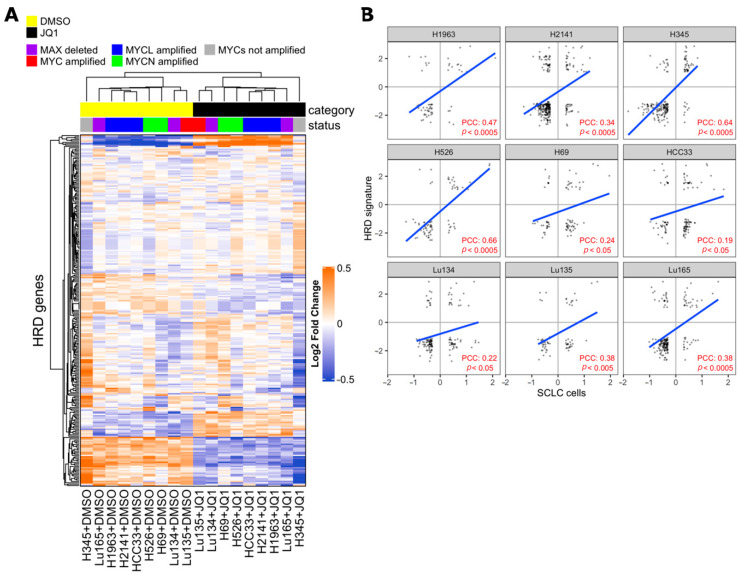
Effect of Bromodomain and Extra-Terminal domain (BET) inhibition on SCLC cells correlates with homologous recombination defect (HRD) signature. (**A**) Heatmap from unsupervised clustering of HRD gene signatures (PMID 24553445) using the gene expression data of SCLC cell lines with or without 1 µM BET inhibitor JQ1 treatment for 24 h of culture (PMID 27764802). JQ1-treated cells formed a cluster with the HRD gene signature. (**B**) Pearson correlation coefficient (PCC) between fold change of HRD genes in SCLC cells (JQ1 vs DMSO) and median fold change of HRD genes in MCF10A^BRCA1kd^, MCFA10^RAD51kd^, and MCF10A^BRIT1kd^ cells vs control cells (HRD signature). Values are expressed in log2 scale. PCC and *p*-values were calculated with R software, cor.test, and lm functions.

Synthetic lethality between PARP inhibition and BRCA1/BRCA2 mutations is well-known and several PARP inhibitors, including Talazoparib, have been approved by FDA as treatments for patients with deleterious germline BRCA-mutated ovarian cancers [26,43]. BRCA1 and BRCA2 proteins are both critical to homologous recombination (HR)-mediated double-strand DNA break repair, supporting the hypothesis that PARP inhibitors are particularly effective on cells with HR defects [26,44,45,46]. Recently, drug synergy between BET inhibitors and PARP-1 inhibitors in breast, ovarian, prostate, and pancreatic HR-proficient cancer cells was reported [47,48,49,50,51,52]. Despite it being concordantly shown that BET inhibition causes defects in the HR-DSBR process, several distinct players of DNA damage response were proposed as targets of BET proteins that mediate loss of homologous recombination function and confer PARPi sensitivity [47,48,49]. In order to determine whether BET inhibition impaired HR in SCLC cells, we applied the HR defect (HRD) gene signature, a selection of 230 genes that were differentially expressed between HR-proficient and HR-deficient cells, to previously published transcriptional profiling data of SCLC cell lines with or without JQ1 treatment [12,49,53]. The expression of the HRD gene signature was coordinately up-regulated or down-regulated in JQ1-treated SCLC cell lines and in HR-deficient cells compared to respective control cells, indicating occurrence of HR defects in SCLC cells by BET inhibition (Figure 4). 

**Figure 5:**
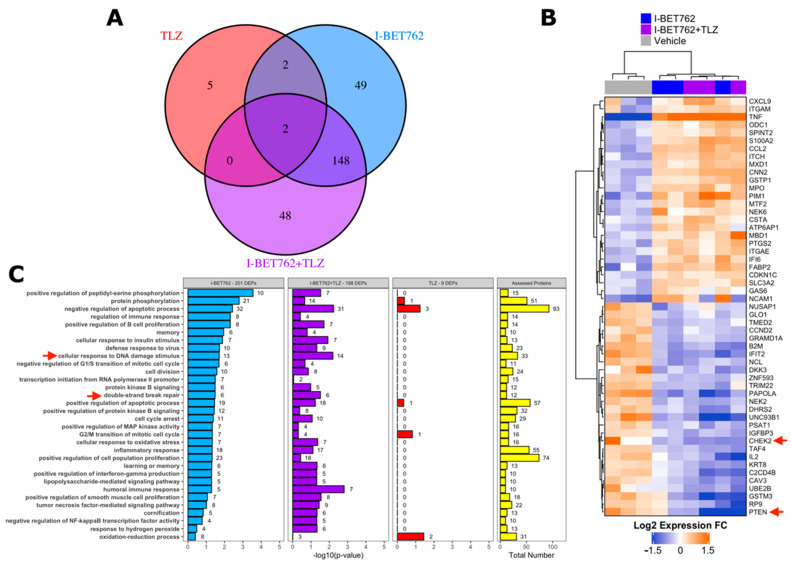
Cancer-related proteome profile of I-BET762- and Talazoparib-treated Lu135 SCLC cells. (**A**) Venn diagram of I-BET762-single agent (I-BET762), Talazoparib-single agent (TLZ) or drug combination (I-BET762+TLZ) associated differentially expressed or phosphorylated proteins (DEPs). DEPs were defined by an expression or phosphorylation level difference of at least 30% between treated and untreated cells (fold change at log2 scale > 0.3785 or < −0.3785 and adjusted *p*-value < 0.05). (**B**) Heatmap of the top-20 down-regulated (blue) and top-20 up-regulated (orange) proteins following I-BET762 or I-BET762+TLZ treatment. The data presented are mean normalized by row. CHEK2 and PTEN (red arrows) are down-regulated in both I-BET762 and I-BET762 treatment groups. (**C**) Pathway enrichment analysis of “biological process” Gene Ontology aspect in DEPs. Ontology classes with Fisher exact test *p*-value < 0.05 in at least one treatment group are shown and ranked based on *p*-value in I-BET762 treatment group.

A proteomic approach was adopted in order to identify the putative molecular mechanism behind the synergistic efficacy between I-BET762 and Talazoparib on *MYCs*-amplified SCLC cells. Levels of 894 cancer-related proteins and their total phosphorylation status were assessed by antibody microarray in SCLC Lu135 cells treated for 24 h with 0.5 µM I-BET762 and 0.5 µM Talazoparib as single agents or in combination [54]. A list of assessed proteins, normalized signal intensities, and protein/phosphorylation fold changes by treatments are shown in Tables S1 and S2. A total of 201, nine, or 198 DEPs were associated with I-BET762 single agent (I-BET762), Talazoparib single agent (TLZ), or combinatorial treatment (I-BET762+TLZ), respectively (Figure 5A). Among the TLZ-associated DEPs, no differentially phosphorylated proteins were detected. The expression levels of TLZ-associated DEPs are shown in Figure S6, and none of them are reported to be associated with SCLC biology or BETi sensitivity. Among the I-BET762-associated and combination-associated DEPs, 150 were common to both treatment conditions (Figure 5A). These common DEPs were equally distributed among each type of alteration, upregulation or downregulation of protein expression or phosphorylation, representing more than 50% of each category (Figure S7A). By plotting the expression or phosphorylation fold changes of these common DEPs in the two treatment conditions one against the other, we confirmed, for all of them, similar upregulation or downregulation degree (Figure S7B). Ranking I-BET762-associated and combination-associated DEPs by their differential expression revealed CHEK2 and PTEN to be among the top-downregulated proteins, whose deficiencies have been associated with HR defects and increased PARPi sensitivity (Figure 5B) [55,56,57,58,59,60,61]. Pathway enrichment analysis was performed while using the biological process aspect of the Gene Ontology. Out of 12,242 biological processes, 305 were represented by at least 10 proteins that were evaluated in the array. However, no significantly enriched classes were identified by multivariate analysis (BH-corrected Fisher exact test *p*-value < 0.05) (Table S3). This is likely a consequence of the limited number of proteins that were assessed in the array, which were chosen based on their association with cancer biology. Indeed, selected ontology classes were enriched of cancer-related ones and numerous assessed proteins belonged to a high percentage of them (Table S4 and Figure S7C). For instance, TNF and TP53, which were DEPs in both I-BET762 and I-BET762+TLZ treatment groups, belonged to 17% and 14% of analysed ontologies, respectively (Table S4). Furthermore, BET inhibition has been reported to affect the expression of thousands of genes, and numerous proteins that belong to distinct molecular pathways are likely to be altered in their expression by I-BET762 [62]. Therefore, we looked at enriched ontology classes before multivariate correction in order to group together DEPs belonging to the most represented pathways in at least one of the treatment conditions (Fisher exact *p*-value < 0.05). “Cellular response to DNA damage stimulus” and “double-strand break repair” were among the mostly enriched ontology classes in both I-BET762- and I-BET762+TLZ-associated DEPs (Figure 5C). Among DEPs that are involved with DNA damage response, CHEK2, FANCC, NBN, OTUB1, UBE2B, and UBE2T have been reported to promote the HR-mediated DSBR process and they were down-regulated in both treatment groups [56,62,63,64,65,66,67,68] (Figure S8). Furthermore, deficiencies in CHEK2, FANCC, or NBN have been shown to induce PARPi sensitivity in other tumor models [56,62,63,64,65,66]. 

Overall, our results indicate that drug synergy between I-BET762 and Talazoparib is associated with the attenuation in HR-DSBR process through the downregulation of various players of DNA damage response by BET inhibition, including CHEK2, PTEN, NBN, and FANCC.

## 3. Discussion and Conclusions

Despite the frequent regression of SCLC tumors after first-line therapy, in the majority of patients the relapse of the disease occurs in few months, leading to a two-year survival rate of less than 15% [1]. Combinations of targeted drugs could be a new potential way of treatment for tumors that are resistant to current pharmacological therapies [23,24].

The identification of the best drug combinations to explore is the first challenge in developing drug combination strategies for treating cancer. Therefore, we took a mixed target-based and a phenotypic approach to identify new drug combinations that, ideally, would be highly toxic in SCLC cells with limited toxicity in non-tumor cells [69,70]. We selected four classes of selective inhibitors whose cytotoxicity has been already shown in SCLC preclinical models and, in some cases, with reported PK properties, and combined them in a mid-throughput screening assay in order to evaluate combinatorial drug efficacy on a panel of SCLC cell lines. Because MYC is frequently amplified or overexpressed in SCLC [4,71], our approach was partially serendipitous and partially target-driven, since we combined two classes of inhibitors that were reported to be effective on cells with elevated levels of MYC proteins with two classes of inhibitors for other oncogene drivers, as PARP-1 and EZH2 [72]. For our purpose, we took advantage of live-cell imaging and time-lapse technology in order to assess the kinetics of drug efficacy, since cytotoxicity that is induced by each single agent could prevent an evaluation of drug synergy with an end-point measurement. Furthermore, we independently assessed the induction of cell death and growth reduction, while taking into account that cytotoxic treatments are ideally more promising than cytostatic ones [73].

We identified a combinatorial drug synergy by BET inhibitor I-BET762 and PARP-1 inhibitor Talazoparib on the suppression of growth and survival of SCLC cells, in particular carrying the amplification of MYC genes, over non-tumor cells. Drug synergy between BET inhibitors and PARP-1 inhibitors in breast, ovarian, prostate and pancreatic HR-proficient cancer cells was recently reported by various independent groups [47,48,49,50,51,52]. Defects in the homologous recombination by BET inhibition has been concordantly shown as the mechanism of action behind drug synergy with PARP inhibitors, but distinct players of DNA damage response were proposed as primary HR-related BET targets that mediate PARP-1 sensitivity. Here, we identified several HR-related proteins that are downregulated by I-BET762 treatment that could, potentially, contribute to drug synergy with Talazoparib in SCLC cells, as CHEK2, PTEN, NBN, and FANCC. Further studies are required in order to assess which of the HR-related proteins are fundamental to inducing defects in homologous recombination and sensitivity to PARP inhibitors in SCLC cells.

In conclusion, these results provide a rationale for exploring new targeted drug combinations for the treatment of SCLC.

## 4. Materials and Methods

### 4.1. Cell Culture

SCLC cell lines HCC33, H69, H2171, and H345 were obtained from Dr. J. D. Minna (University of Texas Southwestern, Dallas, TX, USA), Lu134 and Lu135 from Dr. T. Terasaki (National Cancer Center, Tokyo, Japan), Lu165 from RIKEN BioResource Center (Ibaraki, Japan), H2141 and N417 from the American Type Culture Collection (Manassas, VA, USA), and H446 from the Japanese Collection of Research Bioresources. Human lung fibroblasts MRC5, endothelial cells HECV, and foreskin fibroblasts HFFF2 were obtained from IRCCS University Hospital San Martino—IST National Institute for Cancer Research (Genova, Italy). SCLC cells were cultured in RPMI-1640 supplemented with 10% FBS, 2 mM L-Glutamine, and Antibiotic Antimycotic Solution. MRC5, HECV, and HFFF2 cells were cultured in DMEM high glucose that was supplemented with 10% FBS, 2 mM L-Glutamine, non-essential aminoacids, Sodium Pyruvate 1 mM, and Antibiotic Antimycotic Solution. The cells were cultured at 37 °C, 5% CO_2_ humidified air.

### 4.2. Kinetics of 3D Spheroid Cell Death Assay

Five hundred cells in phenol red-free RPMI that were supplemented with 10% FBS were plated in each well of a 384 multi-well ultra-low attachment plate (Corning, NY, USA, #3830). After 72 to 96 h to allow the formation of homotypic spheroids, JQ1 (Tocris Bioscience, Bristol, UK, #4499), I-BET762 (Sigma-Aldrich Italia, Milan, Italy, #SML1272), Danusertib (Absource Diagnostics, Munich, Germany, #S1107), PF-03814735 (Sigma-Aldrich Italia, Milan, Italy, #PZ0218), GSK126 (Biovision, Milpitas, CA, USA, #2282), Tazemetostat (Medkoo, Morrisville, NC, USA, #406265), Olaparib (Absource Diagnostics #S1060), or Talazoparib (Medkoo, Morrisville, NC, USA, #S204710) were added as single agents or in combination to spheroids, together with CellTox green fluorescent dye (Promega, Madison, Wisconsin, USA, #G8731) for cell death quantification by live-imaging [74]. All of the steps were performed while using Pipetmax automated liquid handling platform (Gilson Italia, Cinisello Balsamo, Italia). Immediately after starting the treatment, spheroids were incubated in Cytation 5 time-lapse cell imaging system at 37 °C and 5% CO_2_ for 24 h (BioTek, Winooski, VT, USA). Images in green fluorescence (GFP led cube) and brightfield were taken with 4× objective immediately after the treatment started and at 4, 8, 12, 16, 20, and 24 h. Subsequently, the plates were moved to a standard incubator and two more readings were performed at 48 and 72 h of treatment. For each reading, three images at distinct Z-stacks covering 100 µm Z-axis were acquired and then merged. The area of spheroid (A) and Green Fluorescence Integral (GFI), delimited by spheroid edges in brightfield, were quantified while using BioTek Gen5 sofware. Spheroid volumes were calculated by the formula 4/3*Area*sqrt(Area/π) [75]. Normalized GFI (NGFI), representative of cell death, was calculated at each time point as GFI/Area. Area under a curve (AUC) was calculated in order to represent overall cell death over time as:

$$\mathrm{AUC}\;=\overset{8}{\underset{i=1}{\sum }}\frac{\left({\mathrm{NGFI}}_{t_{i}}+{\mathrm{NGFI}}_{t_{i-1}}\right)*\left(t_{i}-t_{i-1}\right)}{2}$$

where *t*_1_ = 4 (h of treatment), *t*_2_ = 8, *t*_3_ = 12, *t*_4_ = 16, *t*_5_ i= 20, *t*_6_ = 24, *t*_7_ = 48, *t*_8_ = 72, and *t*_0_ = 0 (immediately after starting the treatment). Highest Single Agent Combination Index (HSA CI) was calculated as:

$$\mathrm{HSA}\;\mathrm{CI}\;=\;\frac{{\mathrm{AUC}}_{AB}}{\max\left({\mathrm{AUC}}_{A},{\;\mathrm{AUC}}_{B}\right)}$$

where AUC*_AB_* is the value that is observed in drug combination-treated samples and max(AUC*_A_*, AUC*_B_*) is the highest value that is observed among the two single agent-treated samples. *A* value > 1 indicates an additive or synergistic efficacy by the two tested compounds [42]. Each assay was conducted in four technical replicates for treated spheroids and sixteen technical replicates for untreated vehicle spheroids (0.05% DMSO). Three biological replicates were performed. Cumulative distribution function (CDF) was used in order to calculate the probability that HSA CI would be less than 1 for values greater than 1, or that HSA CI would be greater than 1 for values that are less than 1 (R, function p.norm). *p*-values from CDF were adjusted for multivariate analysis with Benjamini–Hochberg correction (R, function, p.adjust).

### 4.3. Kinetics of Cell Proliferation Assay

A modification of previously reported kinetics of cell proliferation assay was applied [76]. Five hundred cells in phenol red-free RPMI that were supplemented with 10% FBS and SiR-DNA 0.4 µM (Tebu-bio Italy, Magenta, Italy, #SC007) were plated in each well of a 384 multi-well plate (Corning, New York, NY, USA, #3764). After 24 h, I-BET762 or Talazoparib were added as single agents or in combination to the cells, together with CellTox green dye. Immediately after the beginning of the treatment and at 24, 48, and 72 h, four images for each well in far-red fluorescence (Cy5 led cube), green fluorescence (GFP led cube) and phase contrast were acquired with 4× objective and then merged to cover the entire well. The number of nuclei, stained in far-red fluorescence by SiR-DNA, and the number of dead cells, stained in green fluorescence by CellTox, were automatically counted while using BioTek Gen5 sofware. The number of live cells was calculated by subtracting dead count from nuclei count. The percentage of dead cells was calculated by dividing dead count by nuclei count. Each assay was conducted in three technical replicates and three biological replicates.

### 4.4. Long-Term Tumor Spheroid Growth Assay

The cells were plated in a 384 multi-well ultra-low attachment plate (Corning, New York, NY, USA, #3830) at a concentration of 500 cells (Lu135) or 1500 cells (N417, H69) per well in 45 µL phenol red-free RPMI supplemented with 10% FBS. To allow for the formation of spheroids of ~400 µM diameter, N417 and H69 cells were incubated for three days and Lu135 cells were incubated for five days before the beginning of the treatments. Subsequently, 45 µL of treatment-containing medium was added to a final volume of 90 µL. The spheroids were treated with (I) Talazoparib 5 nM, or (II) I-BET762 ranging from 5 µM to 160 nM as single agent, or (III) combinations of Talazoparib with I-BET762. Two times per week, 45 µL of culture medium was aspirated and then replenished with treatment-containing medium for I-BET762- and Talazoparib-treated spheroids. All steps were performed while using an automated liquid handling platform (Gilson Pipetmax). The spheroid area was monitored after three days of treatment and twice per week with 4× brightfield objective (BioTek Cytation 5). Population doubling time (PDT) was calculated as:

$$PDT\;=\;\frac{t_{i}-t_{0}}{\log_{2}\frac{V_{i}}{V_{0}}}$$

where *t*_0_ and *t_i_* are the initial and final time points, 0 h and 336 h (14 days) respectively, and *V*_0_ and *V_i_* are the initial and final spheroid volumes. A PDT value of 500 was given to samples with PDT < 0 or > 500. Bliss Independence Combination Index was calculated as:

$$\mathrm{Bliss}\;\mathrm{Independence}\;\mathrm{CI}\;=\;\frac{V_{AB}}{V_{A}\;\times V_{B}}$$

where *V_AB_* is the mean relative volume (volume in treated spheroids / volume in untreated spheroids) that is observed in drug combination-treated samples after 14 days of treatment and *V_A_* or *V_B_* are the mean relative volumes in the two single agent-treated samples. *A* value > 1 indicates a synergistic efficacy [42]. Twelve technical replicates were performed.

### 4.5. Cancer-Related Proteome and Phospho-Proteome Profiling

Lu135 proteins were extracted with scioExtract buffer (Sciomics) and protein concentration was determined by BCA assay. The samples were analysed for protein expression levels and phosphorylation levels at serine, threonine, and tyrosine residues with scioDiscover antibody microarrays (Sciomics) targeting 894 different proteins with 1172 antibodies [77]. Each antibody is represented on the array in four replicates. The arrays were blocked on a Hybstation 4800 (Tecan, Mannedorf, Switzerland). Slide scanning was conducted while using a Powerscanner (Tecan, Mannedorf, Switzerland). Spot segmentation was performed with GenePix Pro 6.0 (Molecular Devices, Union City, CA, USA). Raw data were analysed using the LIMMA package (R-Bioconductor) after uploading the median signal intensities. For normalisation, a Cyclic Loss normalisation was applied. For analysis, a one-factorial linear model was fitted with LIMMA, resulting in a two-sided *t*-test or F-test based on moderated statistics. The *p*-values were adjusted according to Benjamini and Hochberg. Three biological replicates were performed. For pathway enrichment analysis, Fisher exact test was performed and adjusted by the Benjamini Hochberg correction.

## Figures and Tables

**Table 1:** Cell lines used in drug screenings assay.

No.	Name of Cell Line	Hystotype	MYC/MAX Gene Familiy Status
1	N417	SCLC	MYC amplification
2	H2171	SCLC	MYC amplification
3	H446	SCLC	MYC amplification
4	Lu135	SCLC	MYC amplification
5	H1963	SCLC	MYCL amplification
6	H2141	SCLC	MYCL amplification
7	H69	SCLC	MYCN amplification
8	Lu134	SCLC	MAX inactivation
9	Lu165	SCLC	MAX inactivation
10	H345	SCLC	-
11	HECV	Umbilical cord	-

**Table 2:** Compounds used in drug screenings assay.

No.	Compound Name	Aliases	Class	Status in Clinical Trials
1	JQ1	JQ-1[+]	BET inhibitor	Not evaluated
2	GSK525762	I-BET762	BET inhibitor	Phase 2
3	EPZ-6438	Tazemetostat	EZH2 inhibitor	Phase 3
4	GSK2816126	GSK-126	EZH2 inhibitor	Phase 1 terminated prior to the completion due to an unfavorable benefit risk profile.
5	AZD2281	Olaparib	PARP-1 inhibitor	FDA approved
6	BMN-673	Talazoparib	PARP-1 inhibitor	FDA approved
7	PF-03814735	-	Aurora Kinase Inhibitor	Phase 1
8	PHA-739358	Danusertib	Aurora Kinase Inhibitor	Phase 2

**Table 3:** Population doubling time (PDT) of SCLC cells. Values are mean ± sd after 14 days of treatment with indicated I-BET762 and/or Talazoparib concentrations. Values are mean ± sd for twelve replicates per treatment.

Cell line	Concentration of I-BET762	Population Doubling Time (h)	
		I-BET762	Talazoparib 5 nM + I-BET762
**H69**	-	52.2 ± 2.3	55 ± 2.8
	0.156 µM	52.2 ± 1.4	57.4 ± 2.9
	0.312 µM	53.7 ± 1.1	58.7 ± 1.3
	0.625 µM	55.2 ± 2	64.2 ± 1.9
	1.25 µM	58.5 ± 1.7	76.1 ± 2.1
	2.5 µM	65.7 ± 2.4	87.6 ± 3.4
	5 µM	76.8 ± 2.7	112.6 ± 5.2
**Lu135**	-	80 ± 4.9	119.5 ± 10.7
	0.156 µM	83 ± 4.8	163.1 ± 28.6
	0.312 µM	84.5 ± 4.8	186.9 ± 29.7
	0.625 µM	94.4 ± 6.7	202.3 ± 14.7
	1.25 µM	110.4 ± 6.6	236.7 ± 16.6
	2.5 µM	122.1 ± 9.9	287.6 ± 46.3
	5 µM	172.2 ± 27.8	344.1 ± 42.7
**N417**	-	77.3 ± 5.1	113.5 ± 24.2
	0.156 µM	86.5 ± 7	128.2 ± 15.1
	0.312 µM	93.7 ± 5.5	150.2 ± 32.7
	0.625 µM	103.8 ± 5.8	179.5 ± 26
	1.25 µM	137 ± 13.2	259.7 ± 45
	2.5 µM	161.4 ± 11.7	262.4 ± 111.8
	5 µM	218 ± 35	262.9 ± 101.3

## Supplementary Materials

The following are available online at https://www.mdpi.com/1422-0067/21/24/9595/s1, Figure S1. Timeplot of I-BET762 and Talazoparib-treated normalized green fluorescence intensity, representative of spheroid death. Values shown mean ± SE. **p* < 0.05, ***p* < 0.01, or ****p* < 0.001 BH-adjusted Combination Index CDF, Figure S2. Timeplot of I-BET762 and Olaparib-treated normalized green fluorescence intensity. Values shown mean ± SE. **p* < 0.05, ** *p* < 0.01, or *** *p* < 0.001 BH-adjusted Combination Index CDF, Figure S3. Timeplot of I-BET762 and Olaparib-treated spheroid relative volumes. Values shown mean ± SE. * *p* < 0.05, ** *p* < 0.01, or *** *p* < 0.001 BH-adjusted Combination Index CDF, Figure S4. Timeplot of JQ1- and Talazoparib-treated normalized green fluorescence intensity. Values shown mean ± SE. * *p* < 0.05, ** *p* < 0.01, or *** *p* < 0.001 BH-adjusted Combination Index CDF, Timeplot of JQ1- and Olaparib-treated normalized green fluorescence intensity. Values shown mean ± SE. * *p* < 0.05, ** *p* < 0.01, or *** *p* < 0.001 BH-adjusted Combination Index CDF, Figure S6. Expression of Talazoparib-associated DEPs in each replicate. ID of the antibodies is indicated in parenthesis since two distinct Abs recognizing MSN protein showed significantly increased signal intensitiy, Figure S7. (A) Distribution of I-BET762 associated and combination associated DEPs among types of alteration. DEPs were grouped based on their association with exclusively one condition or with both of them. (B) Scatter plot of expression or phosphorylation fold changes of 150 common DEPs in I-BET762 and I-BET762+TLZ treatment groups. (C) Scatter plot of percentage of ontologies to which each protein of the array belongs among the 305 analyzed ontology classes (*x*-axis) versus the 12242 ontology classes of the "biological process" aspect (*y*-axis). Each dot represents a protein analyzed in the array, Table S1—normalized signal intensities, Table S2—Log Fold Changes, Table S3—Gene Ontologies, Table S4—Number of gene ontologies per protein.
