# Supplementary material for: BET-Inhibitor I-BET762 and PARP-Inhibitor Talazoparib Synergy in Small Cell Lung Cancer Cells
Source: Int J Mol Sci. 2020 Dec 16;21(24):9595. doi: 10.3390/ijms21249595 (PMC7766292; doi:10.3390/ijms21249595)
Supplement: Supplementary file 1 [file ijms-21-09595-s001.zip › Supplemental Figures.docx]

*
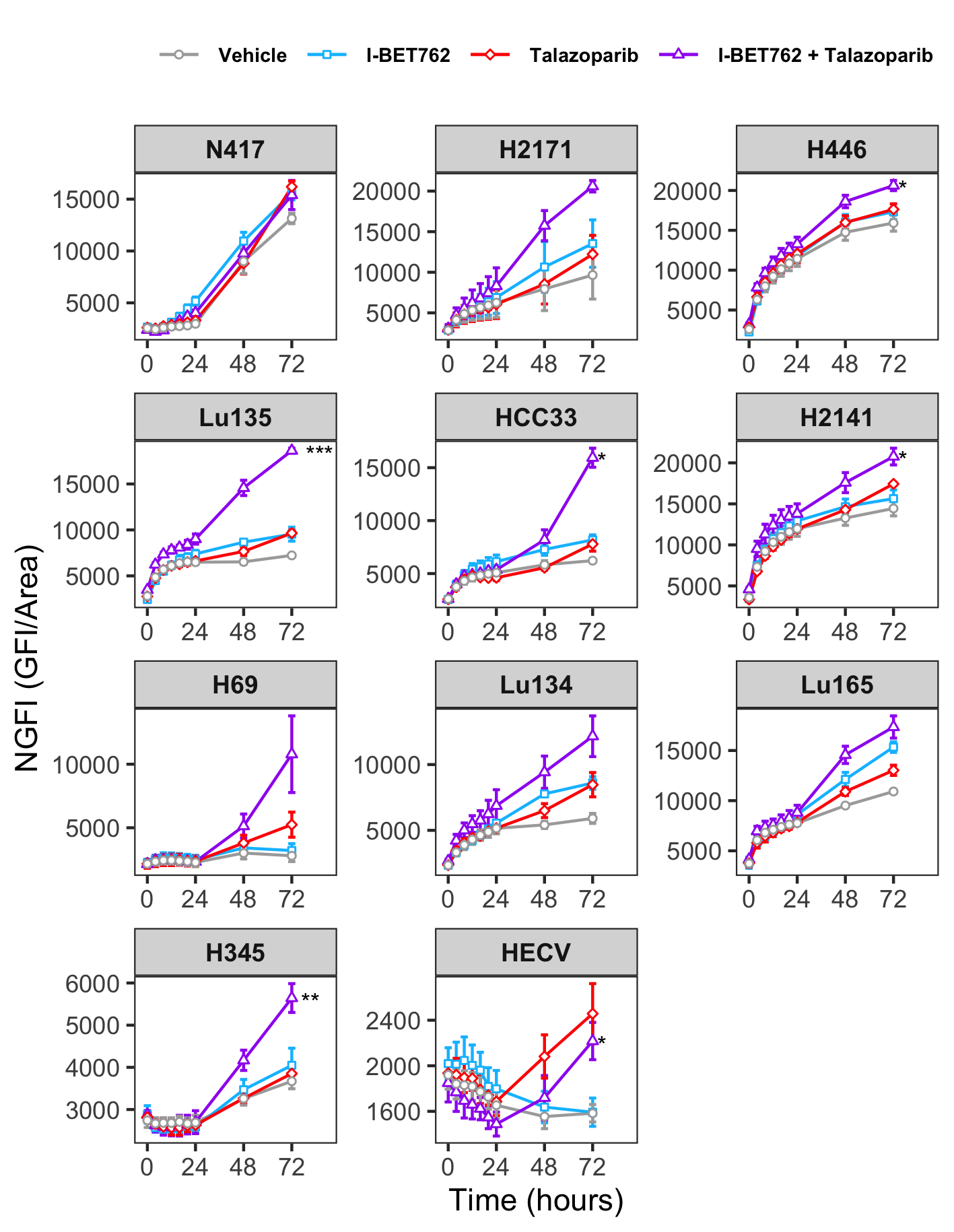
*

**Supplemental Figure S1.** Timeplot of I-BET762 and Talazoparib-treated normalized green fluorescence intensity, representative of spheroid death. *Values shown mean ± SE. *p<0.05, **p<0.01, or ***p<0.001 BH-adjusted Combination Index CDF.*

*
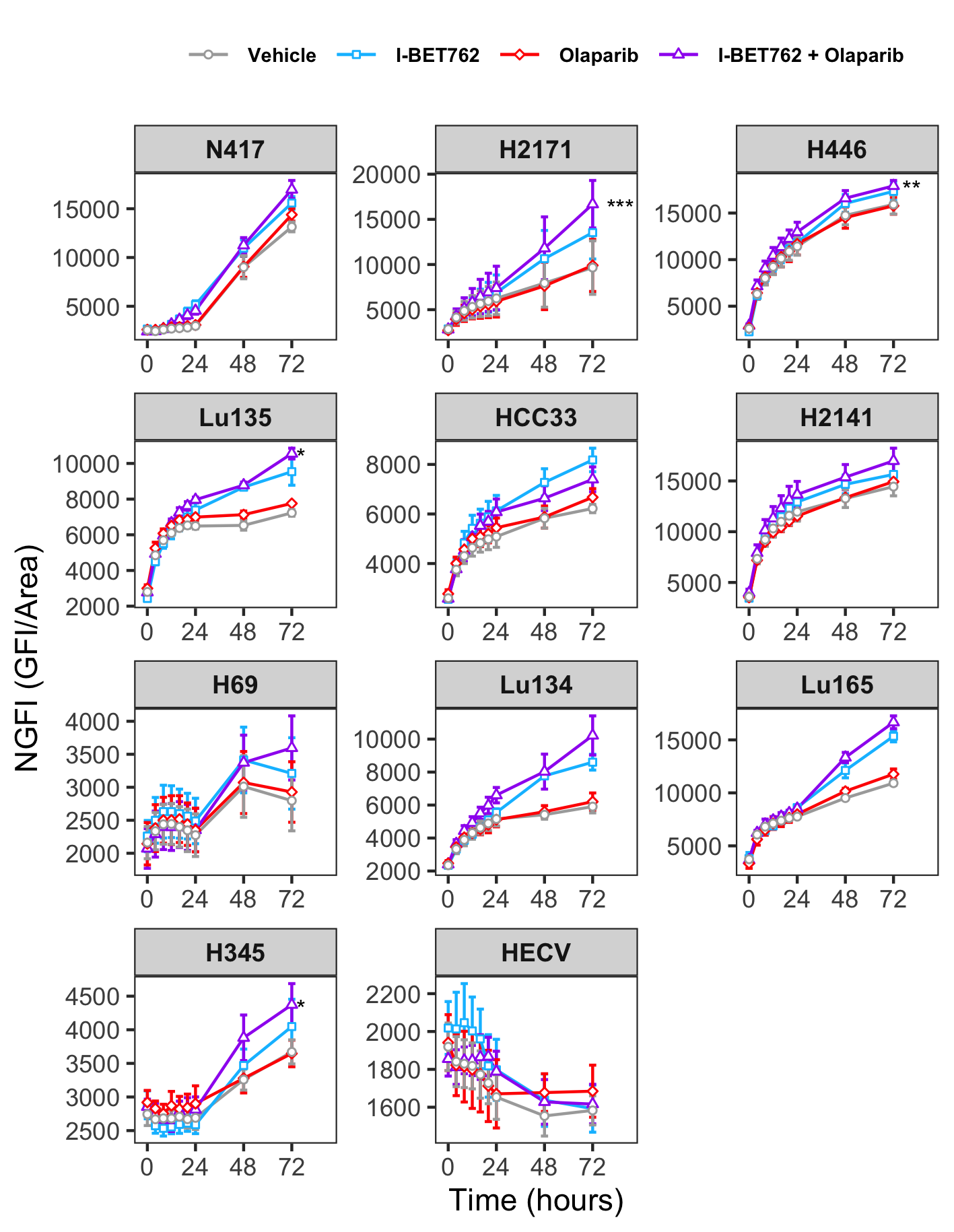
*

**Supplemental Figure S2.** Timeplot of I-BET762 and Olaparib-treated normalized green fluorescence intensity. *Values shown mean ± SE. *p<0.05, **p<0.01, or ***p<0.001 BH-adjusted Combination Index CDF.*

*
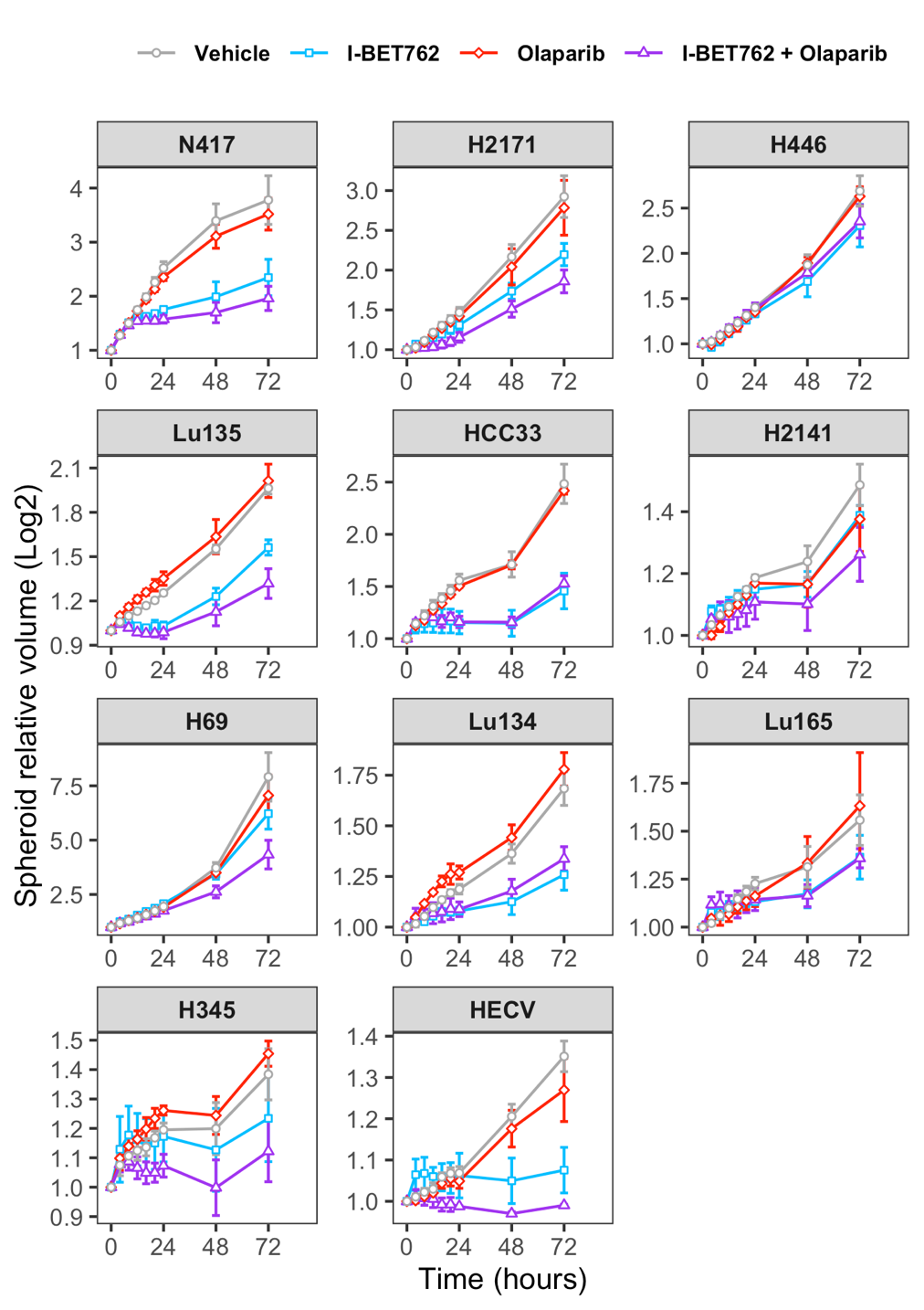
*

**Supplemental Figure S3.** Timeplot of I-BET762 and Olaparib-treated spheroid relative volumes. *Values shown mean ± SE. *p<0.05, **p<0.01, or ***p<0.001 BH-adjusted Combination Index CDF.*

*
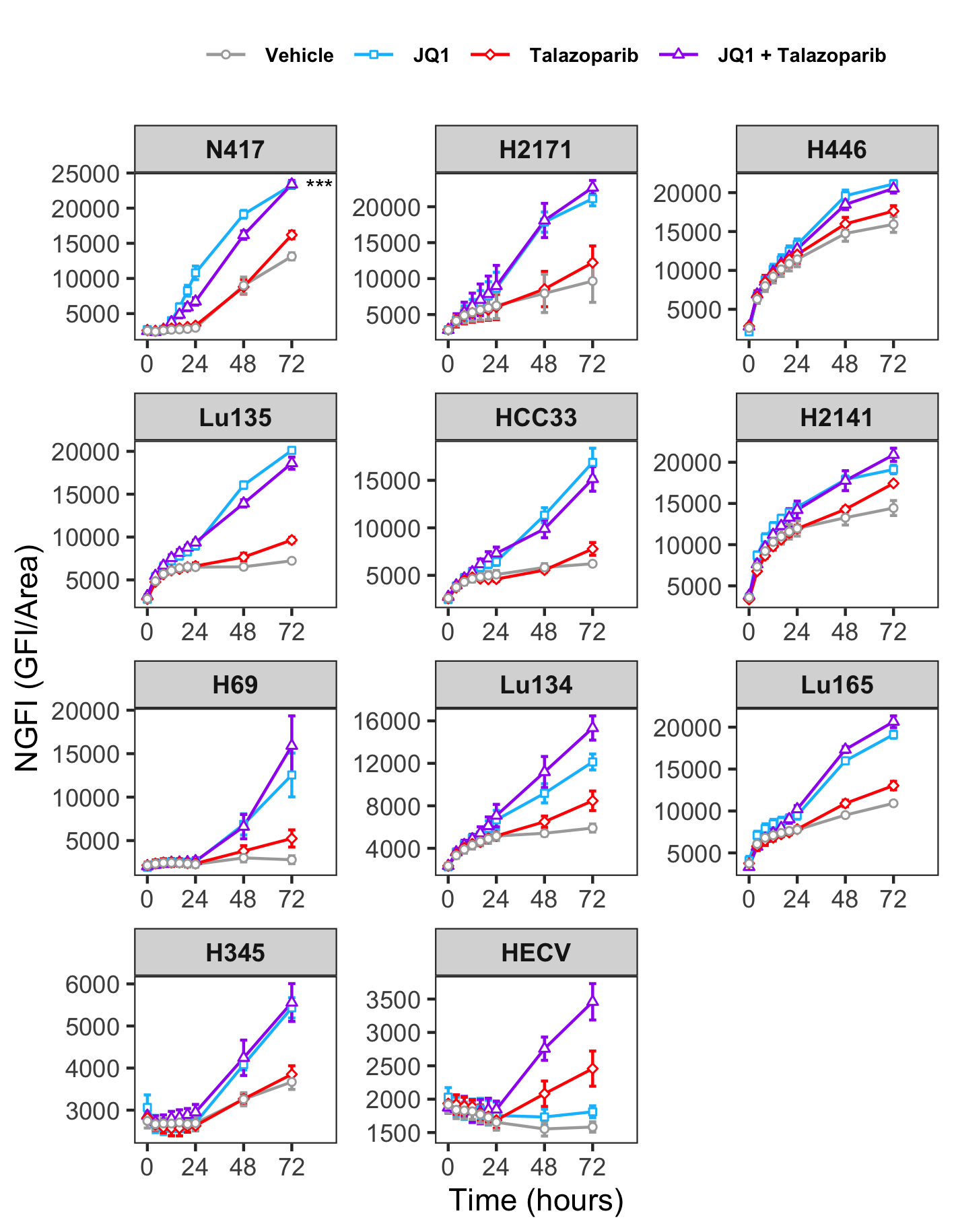
*

**Supplemental Figure S4**. Timeplot of JQ1- and Talazoparib-treated normalized green fluorescence intensity. *Values shown mean ± SE. *p<0.05, **p<0.01, or ***p<0.001 BH-adjusted Combination Index CDF.*

*
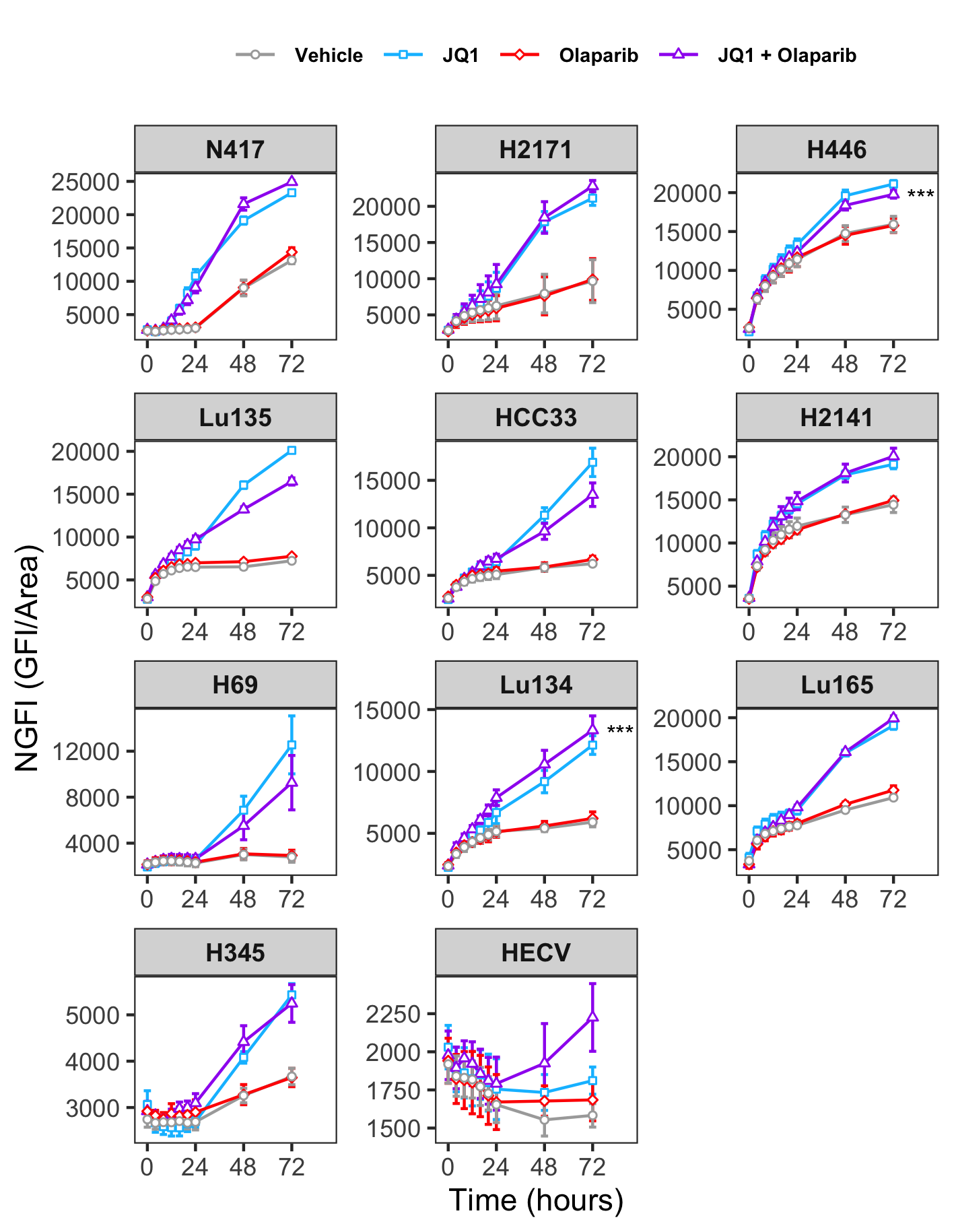
*

**Supplemental Figure S5**. Timeplot of JQ1- and Olaparib-treated normalized green fluorescence intensity. *Values shown mean ± SE. *p<0.05, **p<0.01, or ***p<0.001 BH-adjusted Combination Index CDF.*

*
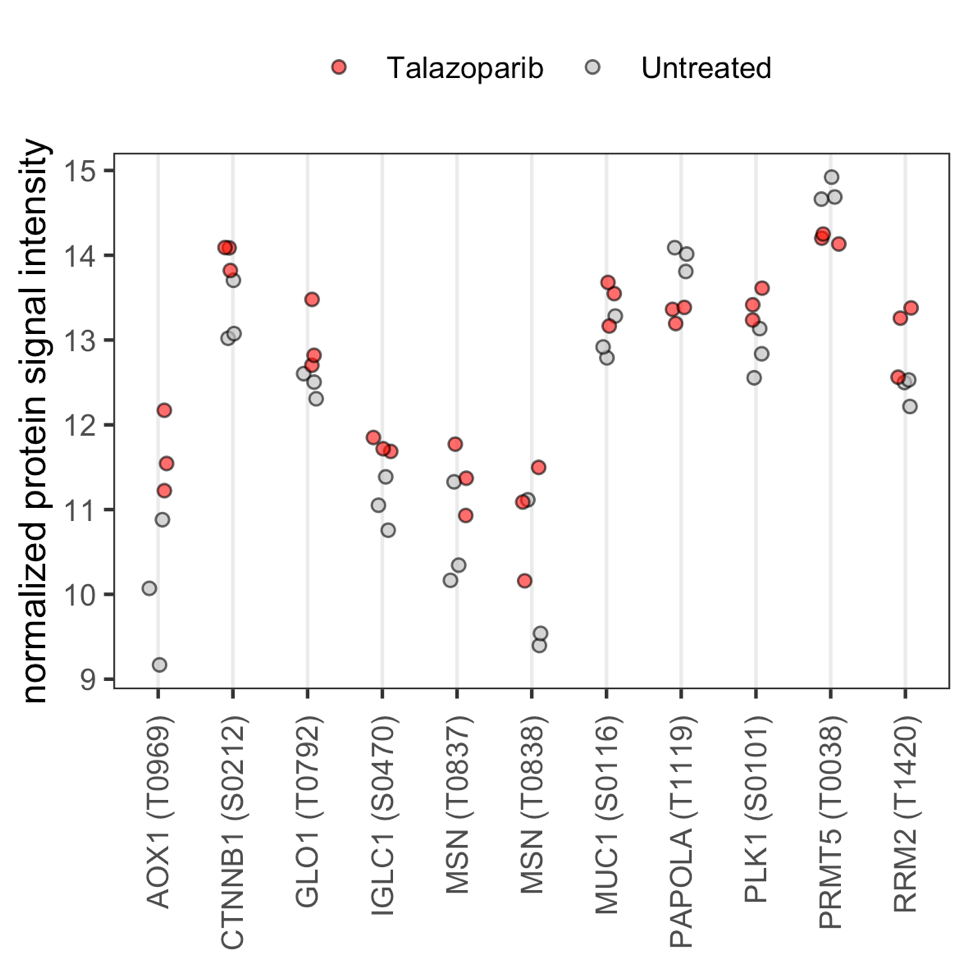
*

**Supplemental Figure S6**. Expression of Talazoparib-associated DEPs in each replicate. ID of the antibodies is indicated in parenthesis since two distinct Abs recognizing MSN protein showed significantly increased signal intensitiy.

*
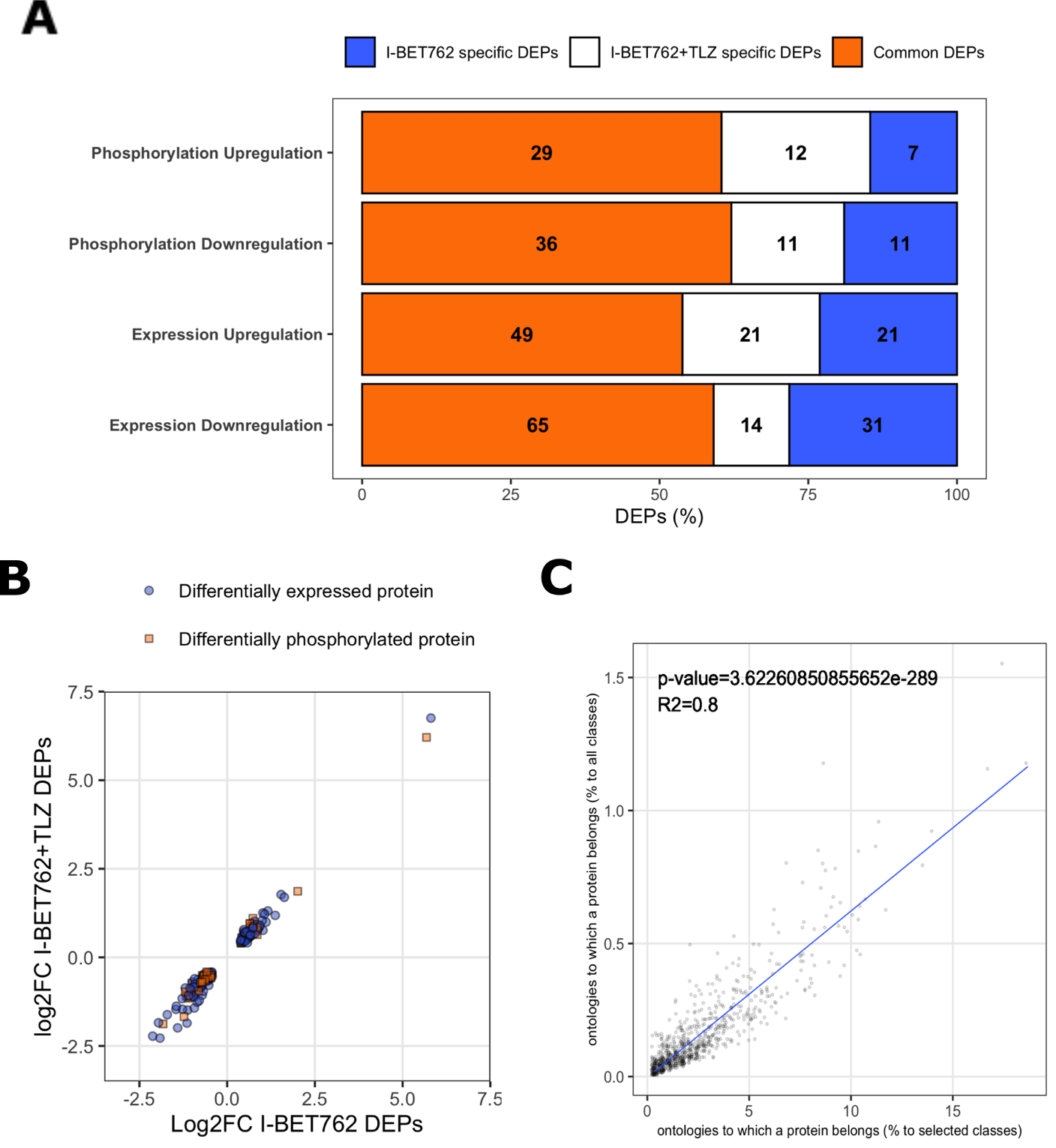
*

**Supplemental Figure S7**. **(A)** Distribution of I-BET762 associated and combination associated DEPs among types of alteration. DEPs were grouped based on their association with exclusively one condition or with both of them. **(B)** Scatter plot of expression or phosphorylation fold changes of 150 common DEPs in I-BET762 and I-BET762+TLZ treatment groups. **(C)** Scatter plot of percentage of ontologies to which each protein of the array belongs among the 305 analyzed ontology classes (x-axis) versus the 12242 ontology classes of the "biological process" aspect (y-axis). Each dot represents a protein analyzed in the array.

*
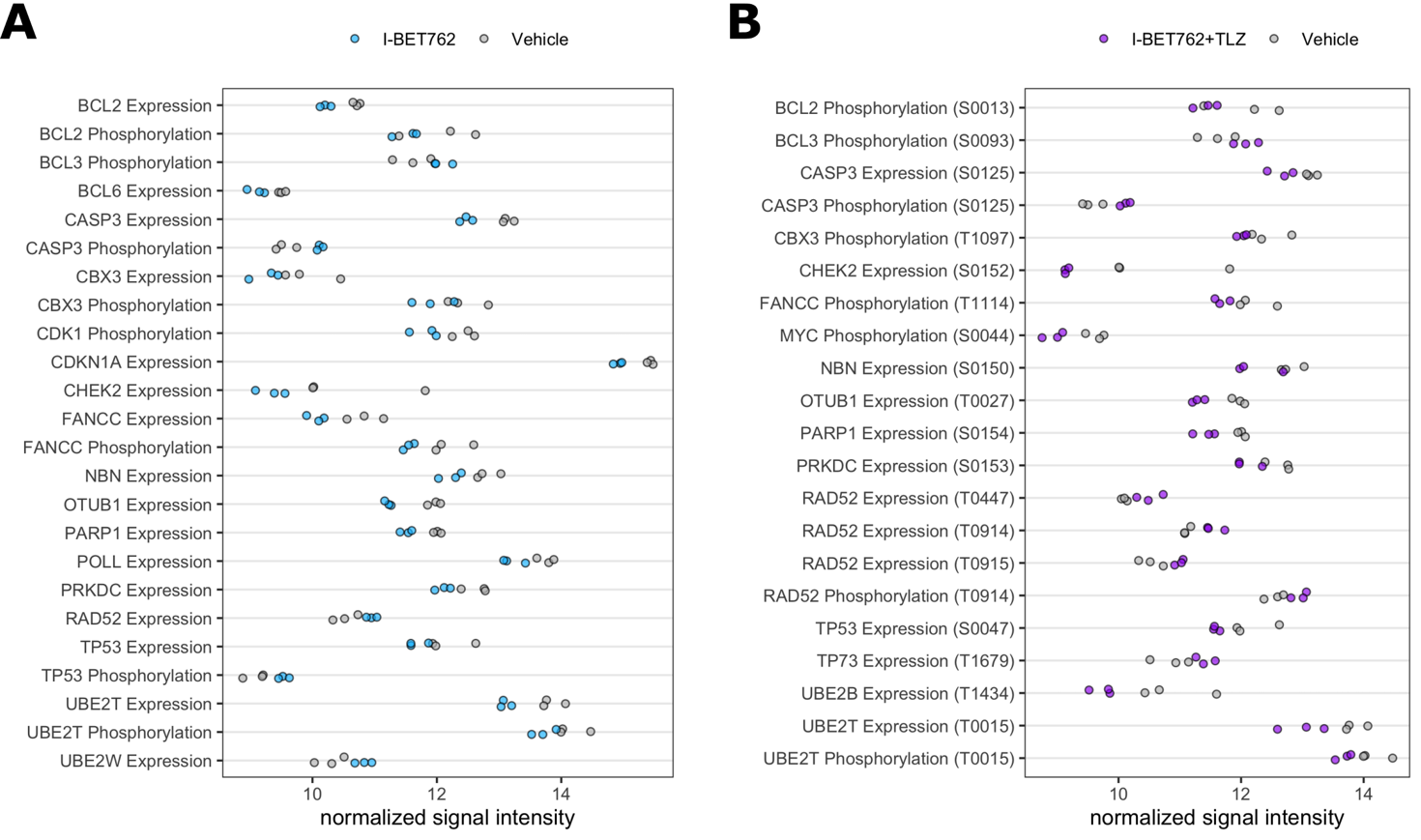
*

**Supplemental Figure S8**. Expression or phosphorylation levels of I-BET762-associated **(A)** or drug combination-associated **(B)** DEPs belonging to DNA damage and double strand-break repair ontology classes in each replicate (GO:0006974, GO:2001021, GO:2001022, GO:2001020, GO:0006281, GO:0045738, GO:0045739, GO:0006285, GO:0006287, GO:0006307, GO:0000731, GO:0006302, GO:0000729, GO:0010792, GO:1903775, GO:0000724, GO:0016924, GO:0000727, GO:0045003, GO:0000730, GO:2000042, GO:1905168, GO:0010569, GO:0006303, GO:2001033, GO:2001034, GO:2001032, GO:0045002, GO:0010792, GO:2000780, GO:2000781, GO:2000779). CASP3, CBX3, CHEK2, NBN, OTUB1, PARP1, PRKDC, TP53, and UBE2T were commonly downregulated in both treatment conditions. ID of the antibodies is indicated in parenthesis in **(B)** since three distinct Abs recognizing RAD52 protein showed significantly increased signal intensity.
